# Does decentralization influence efficiency of health units? A study of opinion and perception of health workers in Odisha

**DOI:** 10.1186/s12913-016-1786-7

**Published:** 2016-10-31

**Authors:** Bhuputra Panda, Harshad P. Thakur, Sanjay P. Zodpey

**Affiliations:** 1 Public Health Foundation of India, IIPH-Bhubaneswar, Bhubaneswar, India; 2 School of Health Systems Studies, TISS, Mumbai, India; 3 Public Health Foundation of India, New Delhi, India

**Keywords:** Efficiency, Decentralization, Rogi Kalyan Samiti, Local decision making, Perception of health workers, Odisha, India

## Abstract

**Background:**

Health systems in low and middle income countries are struggling to improve efficiency in the functioning of health units of which workforce is one of the most critical building blocks. In India, Rogi Kalyan Samiti (RKS) was established at every health unit as institutions of local decision making in order to improve productive efficiency and quality. Measuring efficiency of health units is a complex task. This study aimed at assessing the perception (opinion and satisfaction) of health workers about influence of RKS on improving efficiency of peripheral decision making health units (DMHU); examining differences between priority and non-priority set-ups; identifying predictors of satisfaction at work; and discussing suggestions to improve performance.

**Methods:**

Following a cross-sectional, comparative study design, 130 health workers from 30 institutions were selected through a multi-stage stratified random sampling. A semi-structured questionnaire was administered to assess perception and opinion of health workers about influence of RKS on efficiency of decision making at local level, motivation and performance of staff, and availability of funds; improvement of quality of services, and coordination among co-workers; and participation of community in local decision making. Three districts with highest infant mortality rate (IMR), one each, from 3 zones of Odisha and 3 with lowest IMR were selected on the basis of IMR estimates of 2011. The former constituted priority districts (PD) and the latter, non-priority districts (NPD). Composite scores were developed and compared between PD and NPD. Adjusted linear regression was conducted to identify predictors of satisfaction at work.

**Results:**

A majority of respondents felt that RKS was efficient in decision making that resulted in improvement of all critical parameters of health service delivery, including quality; this was significantly higher in PD. Further, higher proportion of respondents from PD was highly satisfied with the current set of provisions and manners of functioning of the sample health units. Active community engagement, participation of elected representatives, selection of a pro-active Chairman, and training to RKS members were suggested as the immediate priority action points for the state government. Mean scores differed significantly between PD and NPD with regard to: influence of RKS on individual-centric, organizational-centric and patient-centric performance, and the responsibilities to be entrusted with RKS. Absenteeism was strongly associated with satisfaction and local self-governance. Work-related factors, systemic factors, local accountability and patients’ involvement were found to be the key predictors of satisfaction of health workforce.

**Conclusion:**

The understanding on quality improvement strategies was found to be very poor among the health workers. Tailor-made capacity building measures at district and sub-district levels could be critical to equip the peripheral health units to achieve the universal health coverage goals. Work environment, systemic factors and accountability need to be addressed on priority for retention of health workforce. The hypothesized link between efficient local decision making, perception of health workers about efficiency of health units and the health status of population needs further investigation.

## Background

The National Health Policy of India [1] aims at achieving an acceptable standard of health for the population. The country has a long term goal of improving health outcomes through a robust and responsive health system, delivering efficient and high quality health services that are equitable, accessible, affordable and acceptable to the general public [1, 2]. Among the many-fold challenges that the health system encounters today, managing the workforce, developing incentive-based payment mechanisms, and institutionalizing local monitoring will have long-term impact on efficiency of health units [3]. Studies point out that most regions would miss the Millennium Development Goals (MDG) for health because of slow progress in addressing these challenges [4]. Health workers constitute the most critical and dynamic facet of the health system for effective service livery, and therefore performance of the health system is contingent upon success of reforms related to workforce management. In the last decade, state governments have introduced several measures to improve local accountability, enhance skills and build competence of the health workforce as to improve efficiency of health service delivery [5].

Efficiency has several connotations, as it operates through various levels to influence the immediate service productivity and long-term health outcomes. Therefore, a complete analysis of performance also involves the measurement of effectiveness, including attainment of policy objectives [6]. Further, studies suggest that political decentralisation is said to be associated with higher government efficiency among high GDP per capita countries and lower government efficiency among low GDP per capita countries [7]. In India, thus, good quality of local self-governance in health units ought to lead to improved efficiency of service delivery. Our assumption is efficiency affects and is affected by good governance, overall functioning of health units, and satisfaction of health workforce with existing provisions. Consequently, effectiveness and health outcomes, such as, access, quality and appropriateness could be improved.

It may be measured by various proxy indicators ranging from outputs, such as, reduction in absenteeism, to higher outcome/impact level indicators, such as increase in life expectancy per unit of health care and non-health care inputs [8]. Technical Efficiency Score is often used to measure efficiency at facility level: it considers weighted sum of outputs as numerator and weighted sum of inputs as denominator. However, a key challenge in measuring efficiency lies in making the outputs comparable, which vary widely by providers, patients and geographic areas [9–11]. Quantifying risk adjustment of episode-based measures, attributing patient-related outputs to specific providers (inputs), accounting the adjustment measures for multiple providers, and differentiating proprietary grouper methodologies are some of the other challenges [12]. Finally, identification of ‘outputs’ of the health system is a daunting task as it depends on a number of factors, such as, the increase in quality of life, equity and access to services etc. that are difficult to measure [13]. The popularly used data envelopment analysis (DEA) and data stochastic frontier models have had limited success in evaluating efficiency of a variety of primary health care units. Moreover, efficiency score provides a way of differentiating efficient from inefficient production units, but it offers no guidance about the degree of efficiency or inefficiency [13].

Sebastian and Lemma estimated the efficiency of 60 health posts in Ethiopia, considering the number of health workers as inputs and health education sessions as outputs [14]. Studies by Amado and Santos, Marschall and Flessa, Milliken et al. have used consultations, case load, and patient visits, respectively, as output indicators [15, 16]. Kirigia et al. used DEA-based Malmquist productivity index to assess the technical and scale efficiency and productivity change in various settings: they used total number of duty hours as inputs and considered the number of pap smears collected, family planning clinics held, antenatal care and post-natal care sessions held, number of children immunized, etc. as outputs [17–19]. Other studies categorized the outputs into gross indicators, such as, admissions and preventive medical sessions [20, 21].

In Odisha, decentralization as a health sector reform, argued to improve efficiency of health units, was introduced in a phased manner. Rogi kalyan samitis (RKS) were established at health units to foster local decision making which in turn could improve efficiency, service productivity and quality. RKS as local self-governing institutions are composed of political and civil society representatives, health service providers, administrators/managers and other department officials. However, opponents argue that such reforms are often accompanied by profound changes in the way publicly funded services are resourced, and human, financial and material resources are managed [22]. Since the 2000 WHO report, studies have focused on the efficiency of health care systems of industrialized countries [23, 24]. Moreover, almost all studies in the past focused on measuring outputs, but have not been able to examine the inputs and processes from the point of view of key stakeholders [6, 7, 25–27].

Balaguer et al. argued that the likely efficiency gains from enhanced decentralization increase over time [26]. An analysis of opinions of local elites by De Vries indicated that the support for decentralization was more closely related to existing institutional arrangements, and to the degree to which it was expected to influence one’s own position, than to its inherent merits [27]. In India, however, there is scant literature examining the perspectives of the health workers about the influence of RKS on improving efficiency of local decision making, service productivity and quality of services. This study aimed to assess the perception and satisfaction of health workforce about the role and influence of RKS on the functioning of DMHUs at primary and secondary tiers of health service delivery in Odisha. The specific objectives were to a) analyze perspectives of health workers about key public health service delivery components, as seen through the lens of RKS, in priority and non-priority settings; b) examine their self-perceived importance of and satisfaction with various health service provisions; c) compare composite scores between PD and NPD; d) identify predictors of satisfaction at work; and e) discuss suggestions to improve performance.

## Methods

### Concepts

#### Efficiency

Efficiency of health system is defined by the Institute of Medicine (IOM) as “avoiding waste, including waste of equipment, supplies, ideas, and energy’ [28]. Technical efficiency is defined as the ability of a decision making health unit (DMHU) to provide maximum quantities of health services (outputs) from a given set of health system resources or inputs [17].

#### Priority and non-priority districts

Government of Odisha has three administrative zones on the basis of several indices, and each zone covers ten districts. We considered infant mortality rate (IMR) as a proxy of population health; from each zone, the district with the highest IMR constituted priority- and the one with the lowest IMR constituted non-priority district for the purpose of this study.

#### RKS

A registered society established in each health unit, and is governed by the designated government officials, administrators/managers, elected political representatives and civil society organizations. RKS has been assigned with several responsibilities and functions by the central/state government to ensure accountability and transparency in local decision making, improve local responsiveness, efficiency and effectiveness of health services in health units.

#### AYUSH MO

Medical officers from the indigenous streams of Ayurveda, Yoga, Unani, Siddha and Homeopathy disciplines, appointed by the government at different tiers of the health system for provision of health care. They have curative, preventive and promotive roles and are popularly termed as AYUSH medical officers.

#### Composite scores

We converted ordinal data into composite scores, involving several questions/items of the questionnaire on influence of RKS on individual-centric performance, organization-centric performance, future responsibilities to be entrusted with the RKS, perceived importance of individual-and organization-centric performance, and the mean satisfaction score on individual-and organization-centric performance. This technique was used to improve inferences and reduce subjectivity of data analysis.

### Sampling

For primary and secondary tier of health services, the state of Odisha has 6688 sub centres (SC), 1162 primary health centres-new (PHCN), 117 primary health centres (PHC), 231 community health centres (CHC), 22 sub-divisional hospitals (SDH), and 32 district headquarters hospitals (DHH) [29]. We adopted a multi-stage stratified random sampling technique for selection of DMHUs. Using the annual health survey (AHS) report of 2011, districts were ranked against IMR. From each zone, one priority and one non-priority district was selected as the primary sampling units (PSU). All service delivery institutions in the sample districts constituted secondary sampling units (SSU). To avoid the urban-rural bias, from each sample district, the district hospital (DH) was invariably included in the study sample. Two CHCs, one each, from urban and rural areas were included; further, two PHCs, under the administrative jurisdiction of the sample CHCs were selected. Thus, 30 institutions (1 DHH, 2 CHCs and 2 PHCs per district) spread across six sample districts constituted the sites for data collection. Medical officers (MO), Medical officers of indigenous system (Ayurveda, Yoga, Unani, Siddha and Homeopathy) also termed as AYUSH MOs, staff nurses (SN), pharmacists, laboratory technicians (LT), lady health visitors (LHV), male multi-purpose health supervisors (MPHS), and public health extension officers (PHEO) constituted the universe. Taking ‘perception about performance of health units’ as the principal outcome variable, with upper limit of 80 % and lower limit of 70 %, we estimated 61 sample from each category of districts to be adequate to provide 95 % confidence level and 80 % power. Depending upon their availability and willingness to participate, data was collected from a total of 130 health workers.

### Data collection and management

We referred to the ‘WHO health systems building blocks’ framework (2007) for designing a semi-structured, self-administered questionnaire. It contained mostly close-ended questions with multiple choice options, and three open-ended questions to elicit their suggestions about improving local self-governance of health units. The tool was developed in English, field-tested and translated into local language. Two field investigators were hired, trained and engaged in data collection. The primary researcher visited all sample health units, partly collected data, and monitored quality of data collection. Since all the respondents were educated, we thought it appropriate to institute a self-administered tool. In the first section of the tool, basic profile of respondents was captured; in the next section, respondents were asked to rank their perception about the influence of RKS with respect to efficiency in local self-governance, transparency in decision making, community involvement, reduction in absenteeism, improvement in staff motivation and performance, quality improvement, service delivery innovations, and the future responsibilities that may be entrusted with the RKS. In the last section, respondents ranked the importance of and satisfaction with various provisions, such as, infrastructure, funds availability, quality of services, behaviour of co-workers, patients’ involvement, protection of patients’ rights, etc. We used Likert’s 5-point scale (5 = very high and 1 = very low) to rank responses. The study was approved by an independent institutional ethical committee of IIPH-Bhubaneswar; permission was obtained from the health & family welfare department, government of Odisha for data collection. Written, informed consent was obtained from all the respondents prior to administration of the questionnaire. Anonymity of responses was ensured through coding. The respondents were free not to answer some/all questions, and to leave the interview at any point during interview. Data collection was done during Nov 2013–June 2014.

### Data analysis

All data were entered into MS Access 2007 (Microsoft Inc., Redmond, WA, USA), after cleaning and validation. We used SPSS 20.0 (SPSS Inc) and R 3.2.1 for data analysis. The proxy for decentralization was existence of registered RKS - we considered it as an input. Perception & satisfaction of the health workforce, generally considered as process indicators, were our immediate outputs. I) Perception about RKS’ influence was studied by broadly categorizing the health system-related factors into domains of local self-governance, functioning of health units, service delivery and local participation. II) Perceived importance of and satisfaction of respondents with the following provisions were studied in particular: infrastructure, funds availability, quality of services, behaviour of service providers, and community participation. III) For PD-NPD comparison of mean scores, multi-item composite scores were developed on individual-centric performance; organization-centric performance; patient-centric performance; future responsibilities to be entrusted to RKS; importance of individual-and organization-centric performance; and satisfaction with individual-and organization-centric performance. IV) On the basis of regression analysis, predictors of governance and satisfaction at work were identified. Descriptive statistics (mean, median, SD) and linear regression models were used. All continuous variables were described in terms of Mean (+/− SD), Median and their Range. Categorical variables were presented in frequency tables. Comparative analysis of results was carried out to analyze PD-NPD differences. We used Chi square test and independent *t*-test of significance for ordinal and continuous variables, respectively. *P* value of <0.05 was considered as ‘significant’, and of <0.001 as ‘highly significant’. Open-ended responses, on the other hand, mainly contained descriptive suggestions to improve functioning of health units. We undertook thematic analysis of those responses and summarized the key findings.

## Results

### Profile of respondents

A sample of 130 health workers responded to the questionnaire (*N* = 59 in PD and *N* = 71in NPD) which comprised of 63 males and 67 females. The proportion of male and female respondents was evenly matched at 48.5 and 51.5 %, respectively, in PD and NPD (*p* = 0.886). The respondents had a mean age of 43.4 years (SD ± 10.39). There was no significant difference in the mean age of respondents between PD (42.41 ± 11.63) and NPD (44.27 ± 9.25) (*t* = −1.009, *p* = 0.315). However in PD, higher proportion of health workers was < 35 years age (*PD* = 36.2 %; *NPD* = 21.1 %; *p* = 0.006). Forty percent of respondents was MOs/AYUSH MOs, 14.6 % SNs, 13.1 % pharmacists, and the rest 32.3 % respondents comprised of LTs, LHVs, MPHSs, and PHEOs.

### Perception on influence of RKS

#### Local self-governance

About three-fourth (72.4 %; *N* = 130) of total respondents felt that there was remarkable improvement in the infrastructure of the facilities owing to the efforts of RKS (*PD* = 81.4 %; *N* = 59 and *NPD* = 65.2 %; *N* = 71; *p* =0.001). About 76 % of respondents felt that due to influence of RKS staff absenteeism had reduced (*PD* =74.5 %; *NPD* = 48.5 %; *p* = 0.002). With respect to availability of funds, 71.2 % of respondents attributed higher availability of funds to functioning of RKS (*PD* = 84 %; *NPD* = 61.8 %; *p* <0.001). When asked as to whether or not there was any improvement in the overall governance level in the health unit, three-fifth (60 %) of them opined that there was ‘high’ or ‘very high’ level of improvement (*PD* = 74.5 %; *NPD* = 48.5 %).

#### Functioning of health units

About 85 % of respondents felt that RKS positively influenced the motivation of the health workforce deployed in the sample health units. There was no significant PD-NPD difference in this perception (*p* = 0.115). Similarly about 85 % of total respondents opined that due to the influence of RKS there was a remarkable improvement in the work culture of the health units (*PD* = 49.1 %; *NPD* = 24.3 %; *p* = 0.010). Eighty eight percent (88 %) of health workers felt that the performance of health units had improved in last 5 years and that such improvements were owing to the influence of RKS (*p* = 0.007). About three-fourth of respondents viewed that there was ‘high’ or ‘very high’ level of improvement in the satisfaction level of health workers because of the decision-taking abilities of RKS (*PD* = 56.4 %; *NPD* = 28.6 %; *p* < 0.001). Further, 69.3 % of respondents felt that RKS was effective in improving the quality of health service delivery. About one-fifth of respondents ranked the efficiency of RKS as ‘average’, and about 9 % ranked it to be ‘ineffective’. Such perceptions varied significantly between the district categories (*p* <0.001); as compared to PD, higher proportion (85.9 %) of respondents was happy with the manner the RKS was governing the peripheral health units.

#### Service delivery and community participation

In the next section, specific questions were asked as to whether or not the functioning of RKS resulted in improvement of efficiency in health service delivery. Issues, such as, patient waiting time, quality of services, and waste management practices were covered. About three-fourth (75.9 %; *N* = 130) of respondents felt there was reduction of the patient waiting time due to the local responsiveness of RKS (*PD* = 90 %; *NPD* = 66.7 %; *p* <0.001). In NPD, about 31 % of health workers (*N* = 71) felt that there was ‘no change’ in the patient waiting time in last 3 years. About three-fourth of respondents perceived that there was significant improvement in quality of health service delivery due to efficient management of health units by the RKS. About one-fourth of respondents in PD (*N* = 57) and about a tenth of respondents in NPD (*N* = 71), however, felt there was ‘no improvement’ in the quality of services (*p* = 0.051). About 82 % of health workers opined that RKS played crucial role in improving the waste management practices (*PD* = 94.3 %; *NPD* = 72.5 %) as reflected in Table 1. About 62 % of respondents opined that there had been remarkable increase in local accountability and community involvement in last 5 years. The scenario in PD and NPD was distinctly different (*PD* = 81.5 %; *NPD* = 47.1 %; *p* <0.001). About three-fourth of health workers felt that there was strong involvement of community in the functioning of health units (*PD* = 90 %; *NPD* = 65 %; *p* < 0.001).

**Table 1:** Perception about influences of RKS on accountability and participation

Attributes	District Classification						*P* value
	Priority		Non priority		Total		
	No.	%	No.	%	No.	%	
Local accountability increased?							
Very Less (Very Low)	0	0.0	2	2.9	2	1.6	*p* < 0.001
Less (Low)	3	5.6	5	7.4	8	6.6	
Not changed (No Change)	7	13.0	29	42.6	36	29.5	
Much (High)	16	29.6	21	30.9	37	30.3	
Very Much (Very High)	28	51.9	11	16.2	39	32.0	
Total	54	100.0	68	100.0	122	100.0	
Involvement of patients in decision making improved?							
Very Less (Very Low)	1	1.9	0	0.0	1	0.8	*p* < 0.001
Less (Low)	2	3.8	0	0.0	2	1.6	
Not changed (No Change)	2	3.8	24	34.3	26	21.1	
Much (High)	16	30.2	30	42.9	46	37.4	
Very Much (Very High)	32	60.4	16	22.9	48	39.0	
Total	53	100.0	70	100.0	123	100.0	
Patient waiting time reduced?							
Very Less	0	0.0	1	1.5	1	0.8	*p* < 0.001
Less	1	1.9	2	2.9	3	2.5	
Not changed	4	7.5	21	30.9	25	20.7	
Much	18	34.0	30	44.1	48	39.7	
Very Much	30	56.6	14	20.6	44	36.4	
Total	53	100.0	68	100.0	121	100.0	
Average length of stay improved?							
Very Less	1	2.0	0	0.0	1	0.9	*p* = 0.001
Less	2	3.9	2	3.0	4	3.4	
Not changed	2	3.9	15	22.7	17	14.5	
Much	18	35.3	34	51.5	52	44.4	
Very Much	28	54.9	15	22.7	43	36.8	
Total	51	100.0	66	100.0	117	100.0	
Quality of services improved?							
Very Less	0	0.0	0	0.0	0	0.0	*p* = 0.051
Less	1	1.9	1	1.5	2	1.7	
Not changed	6	11.3	19	27.9	25	20.7	
Much	20	37.7	29	42.6	49	40.5	
Very Much	26	49.1	19	27.9	45	37.2	
Total	53	100.0	68	100.0	121	100.0	
Waste management practices improved?							
Very Less	0	0.0	0	0.0	0	0.0	*p* = 0.004
Less	0	0.0	2	2.9	2	1.6	
Not changed	3	5.7	17	24.6	20	16.4	
Much	23	43.4	32	46.4	55	45.1	
Very Much	27	50.9	18	26.1	45	36.9	
Total	53	100.0	69	100.0	122	100.0	

### Importance of and satisfaction with existing provisions

#### Infrastructure, funds and quality of services

In the order of descent, more than 90 % of respondents ranked infrastructure, availability of funds and cleanliness of the institution as the top three important indicators for efficiency measurement of a health unit. Such responses were higher in proportion in the NPD. With regard to their satisfaction with the existing infrastructure, availability of funds, and cleanliness of health units, around 20 % of health workers were ‘highly satisfied’ and the rest 80 % had a different viewpoint. Higher proportion of respondents from PD were satisfied and the range varied from 32 to 36 %, as compared to 9 to 15 % in NPD (*p* < 0.001, 0.002 and 0.001, respectively). Further, about one-fifth of respondents was ‘highly satisfied’ with the performance of service providers, and about 30 % was ‘highly satisfied’ with the quality of services provided to the outpatient (OPD) and in-patient (IPD) cases (Table 2). Higher proportion of respondents from PD were ‘highly satisfied’ as compared to NPD (*p* = 0.001).

**Table 2:** Satisfaction of health workers on governance and quality of services

Attributes	District Classification						Chi^2^, *P* value
	Priority		Non priority		Total		
	No.	%	No.	%	No.	%	
Performance of service providers?							
Least satisfaction	2	4.9	0	0.0	2	1.9	Chi^2^ = 20.802 *p* < 0.001
Less satisfaction	7	17.1	2	3.1	9	8.5	
Average satisfaction	7	17.1	21	32.3	28	26.4	
Often satisfied	11	26.8	34	52.3	45	42.5	
Most satisfaction	14	34.1	8	12.3	22	20.8	
Total	41	100.0	65	100.0	106	100.0	
Quality of services for outpatients?							
Least satisfaction	1	2.2	1	1.5	2	1.8	Chi^2^ = 26.641 *p* < 0.001
Less satisfaction	3	6.7	0	0.0	3	2.7	
Average satisfaction	6	13.3	7	10.8	13	11.8	
Often satisfied	11	24.4	46	70.8	57	51.8	
Most satisfaction	24	53.3	11	16.9	35	31.8	
Total	45	100.0	65	100.0	110	100.0	
Quality of services for in-patients?							
Least satisfaction	1	3.0	1	1.6	2	2.1	Chi^2^ = 20.908 *p* < 0.001
Less satisfaction	3	9.1	0	0.0	3	3.2	
Average satisfaction	6	18.2	6	9.8	12	12.8	
Often satisfied	7	21.2	41	67.2	48	51.1	
Most satisfaction	16	48.5	13	21.3	29	30.9	
Total	33	100.0	61	100.0	94	100.0	
Community participation?							
Least satisfaction	1	2.6	1	1.5	2	1.9	Chi^2^ = 16.046 *p* = 0.003
Less satisfaction	6	15.4	1	1.5	7	6.7	
Average satisfaction	10	25.6	29	44.6	39	37.5	
Often satisfied	12	30.8	29	44.6	41	39.4	
Most satisfaction	10	25.6	5	7.7	15	14.4	
Total	39	100.0	65	99.9	104	99.9	
Transparency in decision making?							
Least satisfaction	3	7.9	0	0.0	3	2.9	Chi^2^ = 19.935 *p* = 0.001
Less satisfaction	3	7.9	3	4.7	6	5.9	
Average satisfaction	8	21.1	23	35.9	31	30.4	
Often satisfied	9	23.7	31	48.4	40	39.2	
Most satisfaction	15	39.5	7	10.9	22	21.6	
Total	38	100.1	64	99.9	102	100.0	
Regularity of holding RKS meetings?							
Least satisfaction	2	5.1	0	0.0	2	1.9	Chi^2^ = 21.227 *p* < 0.001
Less satisfaction	3	7.7	6	9.4	9	8.7	
Average satisfaction	7	17.9	22	34.4	29	28.2	
Often satisfied	11	28.2	31	48.4	42	40.8	
Most satisfaction	16	41.0	5	7.8	21	20.4	
Total	39	100.0	64	100.0	103	100.0	

#### Behaviour of co-workers

About 95 % of respondents viewed that behaviour of medicos and paramedics are very important in the success of health service delivery (*PD* = 85 to 88 %; *NPD* = 98.5 %). Further, about 46 % of health workers were highly satisfied with the behaviour of their co-workers; the corresponding proportion for staff nurse and paramedical staff were 37.4 and 32.7 %, respectively. In PD majority of health workers were fully satisfied with the behaviour of medical and paramedical staff; whereas in NPD, this proportion ranged from 21.5 to 38.5 %. PD-NPD difference was statistically significant (*p* = 0.041, 0.005, and 0.004, respectively).

#### Community participation

Fourteen to twenty-two percent (14 to 22 %) of respondents were ‘highly satisfied’ with the extent of community participation, transparency in decision making, and regularity of holding RKS meetings (*PD* = 25 % to 41 %; *NPD* = 7 % and 11 %; *p* = 0.016, 0.037, 0.023, respectively).

### Comparison of means and composite scores

The mean composite scores of influence of RKS on individual-centric performance was calculated from three items, and was found to be 12.5 (*PD* = 12.92; *NPD* = 12.18) in a scale of 3–15. The mean composite score of influence of RKS on organization-centric performance was 41.41 (*PD* = 44.09; *NPD* = 39.65) in a scale of 10–50, as it contained 10 items. The mean score of the future responsibilities to be entrusted with the RKS was found to be 28.41 (*PD* = 25.81; *NPD* = 30.21) in a scale of 11–33, whereas the mean score on perceived importance of individual-and organization-centric performance was found to be 73.69 (*PD* = 71.45; *NPD* = 74.53) in a scale of 15–75 as it contained 15 items. Lastly, the mean satisfaction score on individual-and organization-centric performance was 58.02 (*PD* = 58.24; *NPD* = 57.93) in a scale of 15–75 (Table 3). Further, an independent *t*-test of significance was conducted with respect to variables, such as, age, length of services, influence of RKS on individual-centric performance, organization-centric performance and patient-centric performance, future responsibilities to be entrusted with RKS, and satisfaction with the functioning of RKS. We found that there was significant to highly significant difference between PD and NPD on all above variables, except age, length of services and satisfaction composite scores (Table 4).

**Table 3:** Means of key composite scores

District Category		Influence of RKS on individual-centric performance	Influence of RKS on organization-centric performance	Influence of RKS on patient-centric performance	Future responsibilities to be entrusted with RKS	Importance of individual- and organization-centric performance	Satisfaction on individual- and organization-centric performance
PD	Mean	12.9273	44.0909	13.3333	25.8125	71.4583	58.2400
	N	55	44	51	48	24	25
	Std. Deviation	2.29213	6.68500	2.16949	6.87783	9.67282	17.24162
PND	Mean	12.1857	39.6567	11.6515	30.2174	74.5313	57.9322
	N	70	67	66	69	64	59
	Std. Deviation	1.85170	5.99887	2.10854	4.49467	3.62517	7.71220
Total	Mean	12.5120	41.4144	12.3846	28.4103	73.6932	58.0238
	N	125	111	117	117	88	84
	Std. Deviation	2.08147	6.61880	2.28506	5.97863	6.01217	11.29343

**Table 4:** *t* test for equality of means between PD and NPD

Independent *t*-test for equality of means							95 % C.I of the difference	
District Category		N	Mean	Std. Deviation	Std. Error Mean	*P* value	Lower	Upper
Age	Priority	58	42.41	11.630	1.527	0.315	−5.491	1.784
	Non priority	71	44.27	9.249	1.098			
Length of service in years	Priority	57	10.25	10.151	1.345	0.662	−2.411	3.785
	Non priority	68	9.56	7.302	.886			
Effectiveness of RKS	Priority	56	3.39	1.056	.141	0.001^a^	−.831	−.215
	Non priority	71	3.92	.692	.082			
Influence of RKS on individual –centric performance	Priority	55	12.9273	2.29213	.30907	0.048^a^	.00799	1.47512
	Non priority	70	12.1857	1.85170	.22132			
Influence of RKS on organization-centric performance	Priority	44	44.0909	6.68500	1.00780	<0.001^b^	2.01956	6.84882
	Non priority	67	39.6567	5.99887	.73288			
Influence of RKS on patient-centric performance	Priority	51	13.3333	2.16949	.30379	<0.001^b^	.89327	2.47036
	Non priority	66	11.6515	2.10854	.25954			
Future responsibilities to be entrusted with RKS	Priority	48	25.8125	6.87783	.99273	<0.001^b^	−6.48705	−2.32273
	Non priority	69	30.2174	4.49467	.54109			
Importance of individual- and organization-centric performance	Priority	24	71.4583	9.67282	1.97446	0.032^a^	−5.87383	−.27201
	Non priority	64	74.5313	3.62517	.45315			
Satisfaction with individual- and organizational-centric performance	Priority	25	58.2400	17.24162	3.44832	0.910	−5.08572	5.70131
	Non priority	59	57.9322	7.71220	1.00404			

^a^Significant; ^b^Highly significant

### Predictors of governance and satisfaction at work

In model 1, we considered decrease in absenteeism as the dependent variable (DV) and governance composite score and satisfaction composite score as independent/intermediate variables (IV). Questions related to role of RKS in local governance in terms of funds flow, information management, logistics management, waste management practices, and quality improvement measures were included in computing the governance composite score. Whereas, satisfaction with human resource management practices, quality of OPD services, behaviour of doctors, cleanliness and decision making process were included to compute satisfaction composite score. Linear regression analysis indicated a highly significant association between the DV and IVs. In model ll, satisfaction composite score was considered as DV and governance composite score as IV; and linear regression revealed a highly significant association between the two (Table 5). In the last section of quantitative analysis, we undertook another linear regression model in which satisfaction composite score (encompassed performance of human resources, quality of OPD services, behavior of doctors, cleanliness of the health units and transparency in decision making) was considered as DV, adjusted with age and gender. Beta coefficient was obtained for IVs, such as, district category, length of service, education level, influence of RKS on a wide range of functions, satisfaction with infrastructure, and with community participation (Table 6). We found significant to highly significant associations between the DV and all IVs except district category, service, education, and funds availability.

**Table 5:** Linear regression of relationship between absenteeism, governance and satisfaction (age and gender adjusted)

Variables	Coefficient (95 % CI)	*P* value
l. Decrease in absenteeism (DV)		
Governance composite score (IV)	.132 (.113 to .152)	<0.001*
SATISFACTION composite score (IV)	.091 (.043 to .139)	<0.001*
ll. Satisfaction composite score (DV)		
Governance composite score (IV)	.267 (.129 to .404)	<0.001*

*Highly significant

**Table 6:** Predictors of satisfaction in the context of local decision making (age and gender adjusted)

Independent variables	Beta coefficient (95 % CI)	*p* value
District category	−.008 (−1.565 to 1.549	0.992
Length of service in years	.052 (−.061 to .166)	0.362
Years of education	−.204 (−.471 to .064	0.134
Staff motivation improved due to RKS?	2.429 (1.403 to 3.455)	<0.001**
Work environment improved due to RKS?	1.881 (.966 to 2.796)	<0.001*
Staff performance improved due to RKS?	2.496 (1.571 to 3.421)	<0.001**
Local accountability increased due to RKS?	.962 (.224 to 1.699)	0.011**
Patient involvement improved due to RKS?	1.908 (1.092 to 2.725)	<0.001**
Governance improved due to RKS?	1.010 (.331 to 1.689)	0.004*
Absenteeism decreased due to RKS?	1.500 (.701 to 2.299)	<0.001**
Fund flow improved due to RKS?	1.176 (.286 to 2.066)	0.010*
Fund availability increased due to RKS?	.742 (−.060 to 1.543)	0.069
Information availability improved due to RKS?	1.794 (.667 to 2.921)	0.002*
Logistics availability improved due to RKS?	1.346 (.407 to 2.284)	0.005*

*Significant. **Highly significant

### Suggestions to improve efficiency

Thematic analysis of open-ended responses indicated that the health workers proposed to actively involve RKS members during programme implementation plan (PIP) preparation. They further suggested to establish locally-monitored supervisory systems as to ensure regularity of RKS meetings and to facilitate continuous community engagement in the management of health units. Involving elected representatives, selecting a pro-active Chairman of RKS, and training to RKS members were cited as key action points for instilling a higher sense of accountability in the local participatory governance institutions in health.

## Discussion

It is now widely accepted that decentralization or shared governance is a difficult concept to define and measure, as it encompasses a wide variety of institutional arrangements and reforms. Similarly, efficiency score has a limited utility in judging the effectiveness of health services. At the same time, past studies have indicated both negative and positive correlations of local governance with its presumed impact on various health outcomes. For instance, Robalino et al. [30] found negative cross-country relationship between decentralization and infant mortality. Zhang and Zou [31] reported negative effect of decentralization on provincial growth in China.

A motivated and committed health workforce is essential for optimal performance of health units. In India, policy makers and implementers of decentralization at DMHU level have paid much attention to the immediate goals of forming and norming the structure and composition of RKS, and fixing their financial envelopes, but the human resource implications of such establishments need a closer scrutiny. It is critical to identify and recognize the elements that make such an arrangement as RKS an exceptionally complex institution of local decision making, especially from the point of view of improving service productivity. In the discourse of decentralization and human resource management (HRM), studies have identified a number of key points that are organized around human resource (HR) planning/staff supply, personnel administration, and performance management. The importance of the management of change is also being highlighted in some studies. There is renewed emphasis to include human resources as a key issue in health system strengthening. Consequently, the concern about the impact of local self-governing institutions on the motivation, satisfaction and performance of health workforce is being increasingly felt. However, global experiences and lessons of different countries on this aspect have not yet been widely shared.

A majority of health workers in our study felt that a positive change had taken place in the governance and quality of service delivery owing to the RKS. Reduction of absenteeism, improvement in local governance, motivation and performance of staff were attributed to RKS, as emphatically perceived by the PD respondents. Similarly, reduction in waiting time, improvement in waste management and local accountability indicate that the health workforce have taken RKS as a desirable instrument in improving efficiency. However, in the absence of a comparable base-line, deriving an inferential causality on this finding goes beyond the scope of this paper. A possible explanation to such findings could be derived out of the general population health (as reflected in the IMR) status, its relationship with efficiency of local decision making process, and perception of the health workforce. Needless to point out here that in the NPD about a third of the respondents didn’t observe any change in the infrastructure; about one-fourth respondents didn’t feel that there was any change in the pattern of staff absenteeism after RKS started functioning; and about two-fifth respondents didn’t observe any improvement in funds availability in last 3 years. Such findings need to be further corroborated with secondary data from these health units.

Infrastructure, funds availability and cleanliness were perceived to be the top three important indicators for efficiency measurement of a health unit. It was also found that higher proportion of respondents from the PD were satisfied with these three existing provisions, as compared to the NPD. Therefore, it was apparent that there was higher need to focus and invest on the part of the government in these three specific areas. Further, a relatively less proportion of respondents were satisfied with the behaviour of their co-workers-this suggests that there is an immediate need for refresher and continued medical education focusing on best client management practices, response time management and responsiveness improvement at work. RKS was perceived to have strong influence on the individual and organizational performance, as reflected in the composite scores which may be reflective of a desirable consequence of RKS’ existence. Further, quality of governance and satisfaction was found to be strongly linked and absenteeism was found to be directly linked to governance and satisfaction. Therefore, any attempt to reduce absenteeism must address governance issues. Therefore, the functioning of RKS and satisfaction of the health workforce with exiting provisions were found to be strongly linked to the overall satisfaction score. Thus, for improved staff satisfaction, the quality of local self-governance must be ensured. However, respondents suggested the government to reconsider modalities of holding meetings, structure and composition of RKS and commitment of the members.

The evidently significant PD-NPD differences in the perception of health workforce could be explained in three possible ways: higher proportion of respondents from PD ranked the influences of RKS as ‘high’ or ‘very high’ in almost all domains except on service delivery, and on importance of infrastructure, funds and cleanliness of health units - this could partly be explained by their low level of understanding and expectation. This could also mean that the health workforce deployed in districts with poor population health status have relatively lower level of expectation from the public health units. Yet another perspective of such an observation could be that the RKS in PD are actively and efficiently discharging the designated responsibilities due to pressing local priorities in view of the poor population health status. Further, there could possibly exist a weak link between RKS functioning and satisfaction of health workforce due to overwhelming effects of other intermediate, process related variables. There is a need to specifically examine the individual characteristics of leadership and socio-political context of districts to find out the reasons for such distinct differences. Higher IMR and poor health indicators on the one hand and positive perception of health workers about RKS functioning, and satisfaction about various provisions of health services on the other, necessitates further probe, in controlled settings, into the level of aspiration, role clarity, and understanding of local priorities of the health workforces across regions.

Reallocation of roles and responsibilities always affects the behaviour of health workforce. After establishment of RKS, local health workers and the managers (often the doctors) are entrusted with more responsibilities to improve the way health services are targeted, organized and managed. To deliver their roles effectively, the workforce needs an appropriate skill mix matching the local needs. In order to foster development of such a strategy, the RKS could need authority for new recruitment and revision of the existing personnel structure [22]. However, very limited powers towards creation of new posts, job enrichment, and human resources management could play as a bottleneck in effective delivery of responsibilities. Our findings confirm earlier literature on low- and middle-income countries (LMIC) that indicate very little evidence that decentralization had resulted in creation of new posts, job re-profiling, or an improved staff mix [32].

Poor quality care during hospital births and lack of healthcare personnel are directly related to mortality reduction and quality improvement measures [33–35]. Local decision making could effectively address local needs, but with certain pre-conditions, such as, good planning, cooperation and informed analysis of processes through a strong information management system [36–38]. However, given that the general understanding about dimensions of quality of health services was poor among the health workforce, this finding could be used by the state government and governments in other similar settings to introduce further reforms around medical and nursing education, local decision making and participatory governance.

## Conclusion

Government may consider investing in infrastructure, funds availability and cleanliness of the health units. Contract management, outsourcing of cleanliness services and cash-flow forecasting are popular strategies to address the existing gaps within the system. The quality of governance need to improve under the constant monitoring and supervision of a state core team as to improve satisfaction and reduce absenteeism. The knowledge base and understanding of health workforce about importance of quality in peripheral health units may be improved through continued education and refresher training. Planning medium-term and long-term skill mix and skill enhancement strategies, supporting research to identify bottlenecks at work and performance monitoring, and sensitizing MOs about the benefits of local decision making process could further improve efficiency of health units. Optimal utilization of existing human resources for health (HRH) could serve as the best medium-term approach for governments that operate challenging environments. Government of India has proposed establishing quality assurance cells at district level units-a step in the right direction. Those cells may be mandated to work closely with the RKS to address the immediate needs of efficient decision making at local levels.

## Limitations

One of the key strengths of this study is its uniqueness in capturing the ranked responses on functioning of RKS, local participation, governance, service delivery components. Representative sample is another strength of this study. Decentralization operates through a wide range of intermediate variables; therefore, studying its impact on perception, opinion and satisfaction of the health workforce was a daunting task. Lack of scaled score-card and analysis among comparable institutions is a limitation. Views about importance and satisfaction of health workers need to be interpreted with caution, as responses could be socially desirable. The link between views of service providers and end-users of services need to be studied further.
